# IKZF3 promotes gastric cancer progression via Hedgehog signaling activation and is targetable by SANT-1: Role of IKZF3 in gastric cancer

**DOI:** 10.3724/abbs.2025103

**Published:** 2025-08-04

**Authors:** Muhammad Ali, Shantanu Baral, Jun Ren, Liuhua Wang, Bin Liu, Sen Wang, Daorong Wang

**Affiliations:** 1 Department of General Surgery Northern Jiangsu People’s Hospital Affiliated to Yangzhou University Yangzhou 225001 China; 2 Northern Jiangsu People’s Hospital Yangzhou 225001 China; 3 Medical College of Yangzhou University Yangzhou 225009 China; 4 Yangzhou Key Laboratory of Basic and Clinical Transformation of Digestive and Metabolic Diseases Yangzhou 225001 China; 5 General Surgery Institute of Yangzhou Yangzhou University Yangzhou 225001 China; 6 Gastric Cancer Centre Department of General Surgery the First Affiliated Hospital of Nanjing Medical University Nanjing 210029 China

**Keywords:** IKZF3, SMO, SANT-1, transcription factor, gastric cancer

## Abstract

Elevated expression of Aiolos family zinc finger 3 (IKZF3), a transcription factor crucial for lymphocyte maturation, is observed in hematological cancers. However, its role in gastric cancer (GC) remains unclear. We detect the increased IKZF3 levels in GC tissues using immunohistochemical, qRT-PCR and western blot analysis. The function of IKZF3 in GC cells is further studied through CCK-8, Transwell, colony formation, scratch wound healing, and flow cytometry assays. *IKZF3* overexpression significantly promotes GC cell invasion, migration, and proliferation, whereas *IKZF3* knockdown induces cell cycle arrest at the G1/S phase. Flow cytometry confirms these alterations in cell cycle dynamics. Using the JASPAR database, we determine that IKZF3 binds to the *SMO* promoter region, thereby activating SMO expression. Notably, the SMO inhibitor SANT-1 effectively reverses IKZF3-mediated effects. Furthermore, IKZF3 promotes GC tumor growth in xenograft models. Our findings highlight the pivotal role of IKZF3 in GC progression by modulating SMO expression and activating the Hedgehog signaling pathway. Therapeutically, targeting IKZF3 with SANT-1 is promising for mitigating GC proliferation and invasion. This study provides insights into potential therapeutic approaches targeting IKZF3 for GC treatment.

## Introduction

Gastric cancer (GC) is a significant universal health issue in East Asian countries [ 1, 2] . In 2020, GC was the third leading cause of cancer-associated death worldwide, with over 1 million new diagnoses and 768,000 deaths reported [3]. By 2021, 26,560 Americans had been diagnosed with GC, and 11,180 passed away from it, ranking it as the sixteen identified cancer and the seventeen leading causes of cancer-related mortality in the USA [4]. Although a small number of special medications have been produced, treating GC patients in the clinic remains extremely difficult because of their unknown fundamental pathogenic mechanisms [5]. To improve patient prognosis, a therapeutic strategy including potent molecular target medications should be considered [6].

The *IKZF3* gene, located at 17q11.22, encodes the transcription factor Aiolos, a key regulator of lymphocyte development and immune function [ 7, 8] . Aiolos, identified as a binding partner of IKAROS, is essential for B-cell proliferation and differentiation. The gene contains eight exons and seven introns. Lenalidomide (Len) targets Aiolos and IKAROS, inhibiting the transdifferentiation of innate lymphoid cell 3 (ILC3) cells to ILC1/NK cells, a process linked to elevated Aiolos expression [9]. Studies in mice lacking Aiolos have revealed its role in Th17 differentiation, B-cell activation, and NK cell maturation [ 10– 12] . Aiolos also collaborates with IKAROS to regulate the expression of λ5, a component of pre-B-cell receptors [ 13, 14] .

Previous studies, by using an integrative approach, analyzed extensive datasets encompassing patient genomics, transcriptomics, and clinical information, which led to the identification of *IKZF3* as a commonly amplified gene in breast cancer with HER2 overexpression [ 15, 16] . Another study indicated a potential role of IKZF3 in the onset and progression of head and neck squamous cell carcinoma [17]. Furthermore, *IKZF3* overexpression has been shown to predict a poor prognosis, particularly in intestinal-type GC. These findings suggest that IKZF3 plays a significant role in the occurrence, metastasis, and prognosis of several neoplasms and hematological malignancies. Therefore, we are interested in exploring the molecular mechanisms that underlie the role of IKZF3 in GC.

Recognizing the molecular etiology of GC is crucial for improving patient survival, as advanced GC is characterized by invasion and metastasis. A deeper understanding of the molecular abnormalities associated with GC pathogenesis can enhance treatment options and improve patient outcomes.

## Materials and Methods

### Bioinformatics analysis

Gene expression and clinicopathological data were analyzed using the UALCAN platform ( uab.edu) to examine TCGA datasets, with survival analysis performed via Kaplan-Meier Plotter ( kmplot.com). Differential expression of IKZF3 in stomach adenocarcinoma (STAD) was validated using GEPIA ( cancer-pku.cn), comparing tumor stages, grades, age, and racial groups. Protein-protein interaction networks and functional pathways were investigated using the STRING database ( string-db.org) to identify biologically relevant associations.

### Cell culture

The human GC cell lines AGS, NCI-N87, and HGC-27 and the epithelial cell line GES-1 were obtained from the Cell Bank of Type Culture Collection of the Chinese Academy of Sciences, Shanghai Institute of Biochemistry and Cell Biology, Chinese Academy of Sciences (Shanghai, China). The cells were routinely screened to ensure that they were free of mycoplasma infection. These cell lines were cultured in RPMI-1640 medium (HyClone, Logan, USA) supplemented with 10% fetal bovine serum (FBS; Life Technologies, Waltham, USA) and 1% penicillin/streptomycin (Solarbio, Beijing, China) at 37°C in a humidified incubator with 5% CO _2_.

### Clinical patient samples

Human surgical specimens, including formalin-fixed paraffin-embedded specimens of GC ( *n* = 80, aged 41–75 years) and freshly excised GC tissues ( *n* = 4), were obtained from Northern Jiangsu People’s Hospital Affiliated with Yangzhou University (Yangzhou, China) between 2022 and 2024. No patient underwent preoperative chemotherapy. The diagnosis of GC was confirmed by two qualified pathologists. Disease staging was determined according to the American Joint Committee on Cancer TNM staging system (AJCC-8 TNM). Clinicopathological data, including age, sex, tumor size, TNM stage, degree of differentiation, histological grade, and venous and nerve invasion, were collected from hospital records. The study was approved by the Northern Jiangsu People’s Hospital Affiliated to Yangzhou University and the Ethics Committee of Yangzhou University. All procedures were conducted in accordance with the ethical standards of the institutional research committee and the Declaration of Helsinki. Written informed consent was obtained from each patient prior to sample collection.

### Quantitative real-time PCR (qRT-PCR)

Total RNA was extracted via TRIzol reagent (Invitrogen, Carlsbad, USA), and 1 μg of the extracted RNA was reverse-transcribed into cDNA via a cDNA synthesis kit (K1622; Thermo Fisher Scientific, Waltham, USA). Each qRT-PCR mixture contained 200 ng of cDNA and was performed with 2× Universal SYBR Green Fast qPCR Mix (ABclonal, Wuhan, China). The amplification protocol consisted of initial denaturation at 95°C for 3 min, followed by 40 cycles of 95°C for 5 s and 60°C for 30 s. The 2 ^–ΔΔCt^ method was used to analyze the target gene expression. The primer sequences are listed in Supplementary Table S1.

### Immunohistochemistry

The clinical prognosis associated with IKZF3 expression was assessed using immunohistochemistry (IHC). The sections were deparaffinized in xylene, rehydrated with a graded alcohol series and citrate buffer, and blocked with 3% hydrogen peroxide. They were then treated with an IKZF3-specific primary antibody (1:50, #15103; CST, Danvers, USA), followed by a biotin-conjugated secondary antibody (Boster, Wuhan, China) and a streptavidin-peroxidase complex.

Slides were examined, and high-power fields (200× and 400× magnification) were randomly selected and imaged with an Olympus BX53 fluorescence microscope (Olympus, Tokyo, Japan). IKZF3 expression was scored on the basis of staining intensity (0 = negative, 1 = weak, 2 = moderate, and 3 = strong) and the percentage of positive cells (0 = 0%–5%, 1 = 6%–25%, 2 = 26%–50%, 3 = 51%–75%, and 4 = >75%). The final staining score was calculated by multiplying the staining intensity by the percentage of positive cells. A cut-off value of 5 was used to classify IKZF3 expression as low (score ≤ 5) or high (score > 5) on the basis of receiver operating characteristic (ROC) curve analysis.

### Western blot analysis

The cells were lysed using RIPA buffer (Beyotime, Shanghai, China). After the protein concentration was determined, 30 μg of protein from each sample was separated by SDS-PAGE and then electro-transferred onto 0.45-μm polyvinylidene difluoride (PVDF) membranes (Millipore, Billerica, USA). The membranes were washed three times with TBST, blocked with 5% skim milk for 2 h at room temperature, and incubated overnight at 4°C with a primary antibody. After being washed three times with TBST, the membranes were incubated with a horseradish peroxidase-conjugated secondary antibody (1:5000; ABclonal) for 2 h at room temperature. Images were captured using an automatic chemiluminescence image processing system (TANON, Shanghai, China). Band intensities were quantified via ImageJ and analyzed using GraphPad Prism 8. The primary antibodies used are listed in Supplementary Table S2.

### Cell transfection

Lentivirus was used to generate stable *IKZF3*-knockdown GC cell lines, namely, NCI-N87 and HGC-27. Overexpression (OE) vectors for *IKZF3* were used in the GC cell line AGS. The following recombinant lentiviral vectors were constructed: negative control sequence, sense, 5′-AATTCTCCGAACGTGTCACGT-3′ and antisense, 5′-ACGTGACACGTTCGGAGAATT-3′; sh-IKZF3, sense, 5′-CCGUCAAAGUGAUCAACAATT-3′; and antisense, 5′-UUGUUGAUCACUUUGACGGTT-3′ obtained from General Biol (Nanjing, China). A 24-well plate with 1 × 10 ^4^ cells was seeded into individual wells 12 h prior to virus infection. After pre-screening with puromycin to identify stable cell lines and evaluating the fluorescence signal in the cells via fluorescence microscopy, lentivirus was given to each well individually and incubated for 72 h. All the cells were grown at 37°C with 5% CO _2_ in a cell incubator.

### Cell Counting Kit-8 (CCK8) assay

A single-cell suspension was prepared by digesting transfected cells with 0.25% trypsin after they were washed twice with PBS once the cells reached 80% confluency. For the experiment, six parallel wells were set up in a 96-well plate, with 1000 cells seeded per well in 100 μL of medium. At time intervals of 24, 48, 72, and 96 h, 10 μL of CCK-8 solution (CK13; Dojindo, Shanghai, China) was added to each well and then incubated for 2 h at 37°C to evaluate proliferation. The optical density (OD) in individual wells was then analyzed using a microplate reader (SkanIt RE 7.0; Thermo Fisher Scientific) at a wavelength of 450 nm.

### Colony formation (proliferation assay)

To assess the colony-forming ability of the cells, 500 cells per well were seeded in a 6-well plate with RPMI-1640 medium supplemented with 10% FBS. The cells were cultured for 14 days at 37°C. The cells were subsequently washed, fixed in 4% paraformaldehyde, and stained with 0.5% crystal violet in 3% acetic acid (v/v) for 20 min at room temperature. Subsequently, images were captured and the number of colonies was quantified under a light microscope (CKX53; Olympus).

### Wound healing assay

Wound-healing assays were conducted to evaluate the migratory ability of the cells. The transfected cells were placed in six-well plates and continuously incubated until a uniform monolayer of cells covered the bottom of the plate. A micropipette tip was then used to create a controlled scratch wound on the cell surface, which was performed slowly and uniformly. Subsequently, the cells were washed multiple times with PBS to eliminate any floating cells. The remaining cells were cultured in RPMI 1640 without FBS. Wound images were captured at 0 and 24 h using an inverted microscope (CKX53). These images were subjected to analysis using ImageJ software (v1.54d).

### Migration and invasion assays

The cells were suspended in serum-free medium with a 24-h starvation period at 48 h after transfection and then washed twice with PBS. The cell density was set to 1 *×* 10 ^4^ cells/mL with serum-free RPMI medium. Three chambers in each group of 24-well 8-μm Transwell chambers (Corning, New York, USA) were used, and 200 μL of cell suspension was added to each chamber. After 600 μL of RPMI-1640 culture medium supplemented with 10% FBS was added to the lower chamber, the cells were incubated at 37°C with 5% CO _2_. For the migration assay, after 48 h, the cells were fixed with 4% paraformaldehyde for 30 min. The chamber was then incubated in a 0.2% Triton X-100 solution (Sigma, St Louis, USA) for 15 min, followed by staining with 0.05% gentian violet for 5 min. The labelled cells were counted under an inverted microscope (CKX53). For the invasion assay, the inserts were pre-coated with 50 μL of Matrigel (Sigma), and the same methods were then employed for fixation and staining as the cell migration assay. The cells were counted randomly in five chosen fields, and the average number of cells was calculated.

### Flow cytometry

The cells were washed twice with PBS before being digested with trypsin. The samples were then fixed in 70% ethanol for 4 h at 24°C. The cells were subsequently incubated for 30 min at 37°C in 500 μL of propidium iodide (PI) staining solution (Beyotime). Cell cycle analysis was performed using a FACS flow cytometer (BD Biosciences, Franklin Lakes, USA), and the data were analyzed with ModFit LT ^TM^ V5.0.9 software (Win 10).

### Chromatin immunoprecipitation (ChIP) assay

ChIP assay was performed using the Simple ChIP Plus Enzymatic Chromatin IP kit (magnetic beads) (#9005S; CST, Danvers, USA). The cells were fixed with 1% paraformaldehyde for 10 min, followed by the addition of 0.125 M glycine to stop DNA-protein crosslinking at room temperature for 5 min. SDS lysis buffer containing protease inhibitors was used to lyse the cells, and chromatin fragments were generated using an ultrasonic fragmentation device. A portion of the lysates was designated as “Input.” The remaining lysates were incubated with anti-IKZF3 antibody (#15103; CST) and Protein G magnetic beads to form a DNA-antibody-magnetic bead complex. After elution and purification, the DNA was labelled “Target”. Rabbit IgG (CST) was used as a negative control. The final purified DNA fragments were analyzed by qRT-PCR. The sequences of the primers used for qPCR targeting the *SMO* promoter binding site are provided in Supplementary Tables S3 and 4.

### Xenotransplantation experiments

A total of 24 male BALB/c nude mice, aged 4 weeks and weighing 18–22 g, were purchased from GemPharma Tech (Nanjing, China). Throughout the experimental period, the mice were housed in a laminar flow chamber free of pathogens. They had unrestricted access to food and water, were maintained under a 12/12-h light/dark cycle, and were maintained at a regulated humidity of 30%–40% and a constant temperature of 25°C. Yangzhou University’s Ethics Committee for Studies on Animals (Yangzhou, China) approved this study (No. 202303827). The Laboratory Animal Guidelines for the Ethical Review of Animal Welfare were followed when the experiments were performed (GB/T 35892-20181).

NCI-N87 cells were transfected with shRNA targeting IKZF3 or the corresponding scramble control via lentiviral infection. Male BALB/c nude 4-week-old mice were assigned to five groups: shCtrl ( *n* = 6), shIKZF3 ( *n* = 6), NC ( *n* = 4), OE-IKZF3 ( *n* = 4), and SANT-1 ( *n* = 4) groups. NCI-N87 cells (1 × 10 ^6^ cells stably transfected) were injected subcutaneously into the mice under the influence of isoflurane inhalation anaesthesia (1%–2%) in their left armpit.

The health and behavior of the mice were monitored every two days to determine whether there were any problems with drinking or feeding, discomfort not being relieved, or distress that did not end. Every week, the tumor volume (V) was determined via the following formula: V = (length × width ^2^)/2. All the mice were sacrificed by cervical dislocation under anaesthesia four weeks post-inoculation. CO _2_ asphyxiation was utilized to anaesthetize the mice (CO _2_ was injected into the chamber at 40%–70% of the chamber capacity per minute to minimize discomfort). The next step was to confirm death via dilated pupils. The tumors were then removed and weighed.

### Statistical analysis

Statistical analysis was performed with GraphPad Prism (version 8.0; GraphPad Software, La Jolla, USA). The quantitative data are presented as the mean ± SD. Student’s *t* test was used to evaluate differences in means between two groups, and Dunnett’s test was used in ordinary one-way ANOVA to analyze differences among several groups. The area estimation and counting were performed with ImageJ (1.46r). The significance level was set at *P*  < 0.05.

## Results

### IKZF3 is overexpressed in STAD according to TCGA datasets and Kaplan-Meier plots

**Figure 1:**
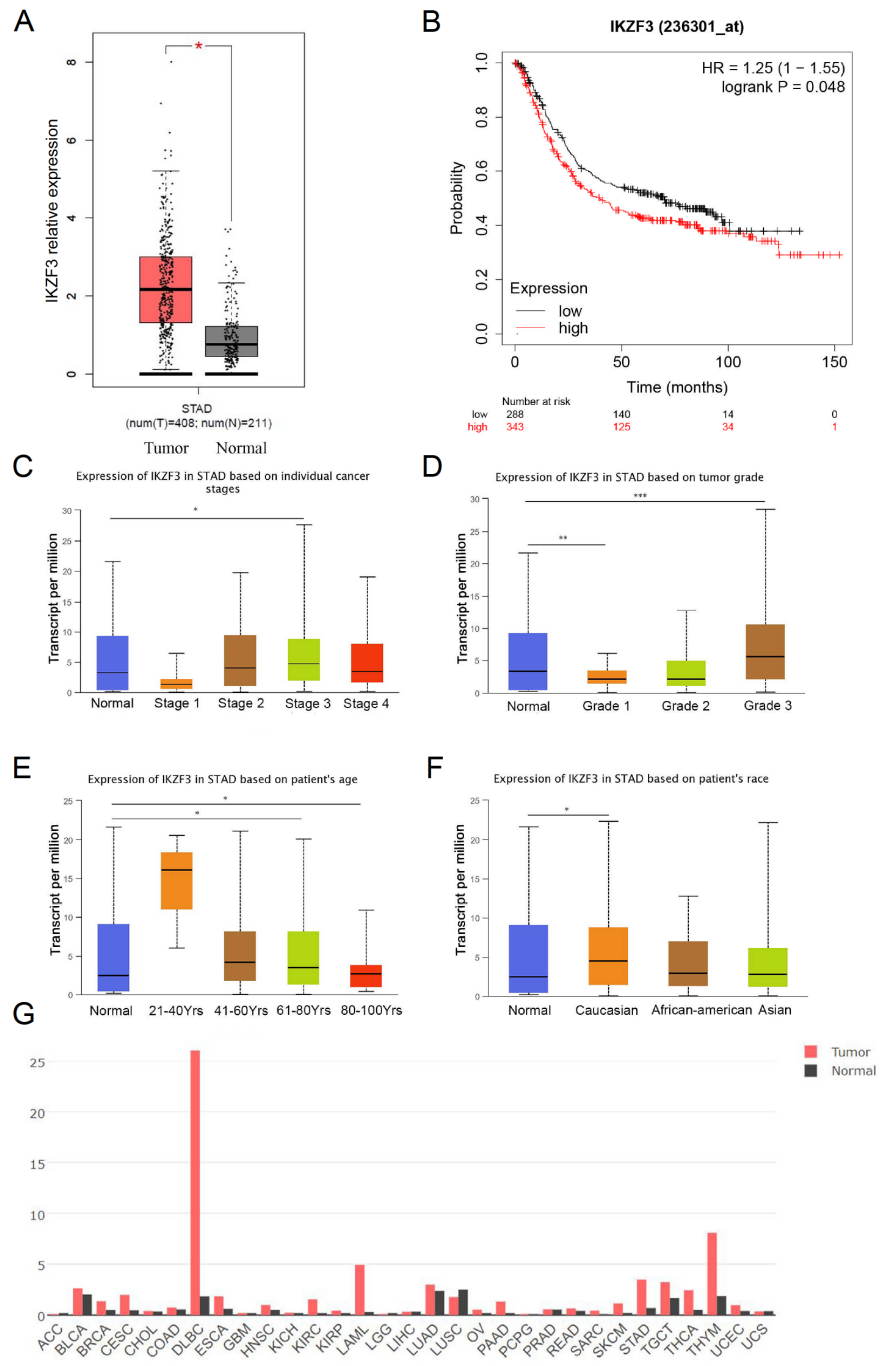
Figure 1 IKZF3 expression is elevated in STAD (A) IKZF3 mRNA expression was assessed in GC samples (n = 408) and normal samples (n = 211) via the online tool GEPIA. (B) Overall survival analysis via Kaplan-Meier plotter revealed significant results (***P < 0.01). (C) IKZF3 expression in stage 3 STAD was significantly different from normal tissue (*P < 0.05), unlike in other stages (D) IKZF3 expression in STAD was significantly higher in grade-1 and grade-3 tumors than in normal tissue (**P < 0.01 and ***P < 0.001, respectively). (E) IKZF3 expression in STAD was significantly higher in patients aged 61–80 years and 80–100 years than in normal tissue (*P < 0.05). (F) IKZF3 expression in STAD was significantly higher in Caucasian patient’s than in normal tissue (*P < 0.05) (G) The gene expression profile was analyzed across all tumor samples and paired normal tissues via GEPIA.

Gene and clinicopathological information was collected from UALCAN, which examines TCGA data online ( Figure 1A). The overall survival graph showed significant results in the Kaplan-Meier plotter ( Figure 1B). Additionally, IKZF3 was found to be overexpressed in stage 3 stomach adenocarcinoma (STAD) compared with normal tissue. Significant differences were also observed in tumor grades 1 and 3, as well as in the age groups 60–80 years and 80–100 years, and within the Caucasian race ( Figure 1C–F). The gene expression profiles across all tumor samples and paired normal tissues were analyzed using GEPIA ( Figure 1G).

### IKZF3 in gastric cancer cell lines and human gastric cancer tissues

**Figure 2:**
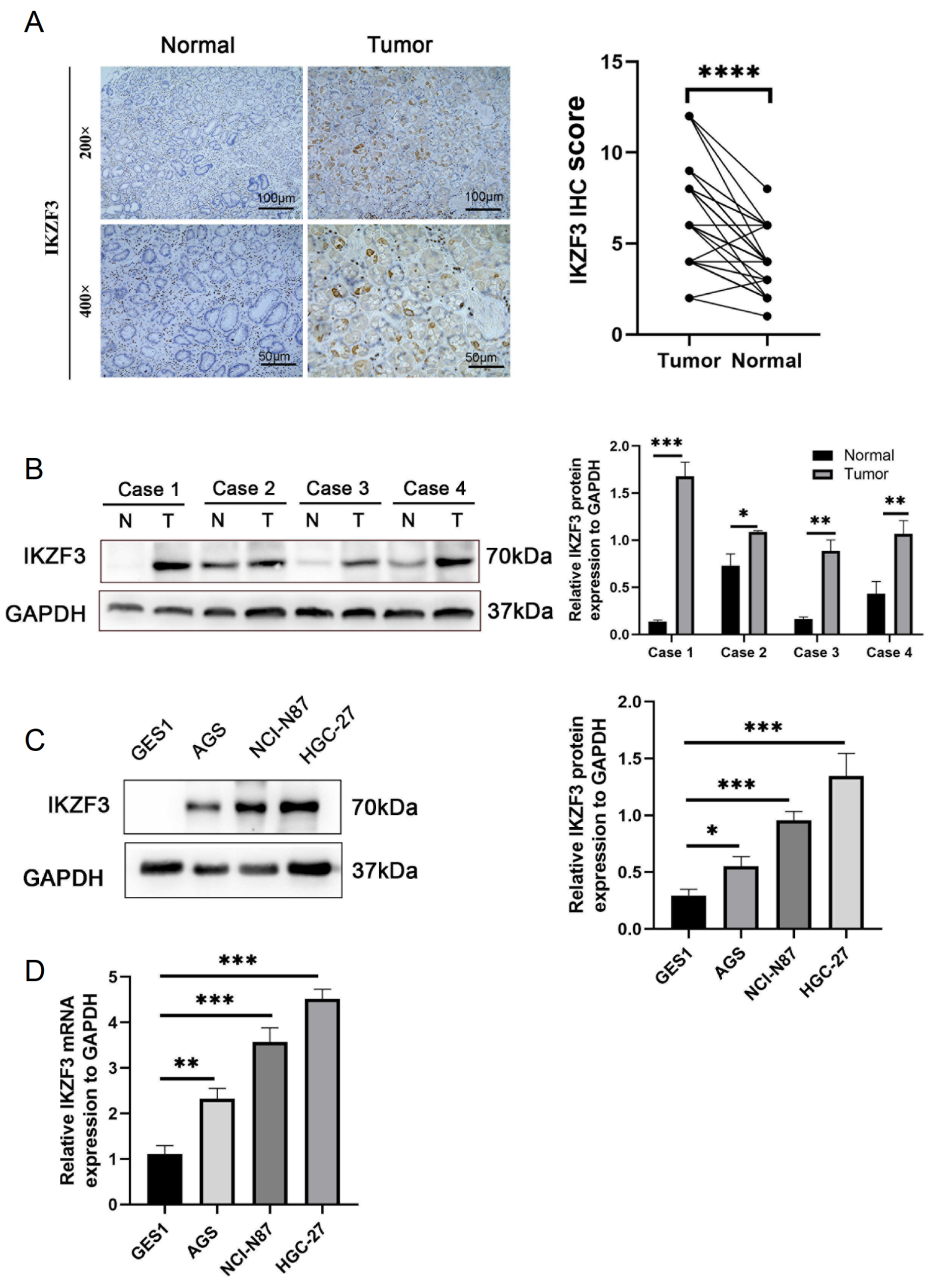
Figure 2 IKZF3 expression analysis (A) IHC analysis of IKZF3 was conducted on noncancerous paired tissue and GC tissue from patients. The scale bar indicates 100 μm at 200× magnification and 50 μm at 400× magnification. ****P < 0.0001. (B) The protein expression levels of IKZF3 in normal and adenocarcinoma human tissues (*P < 0.05, **P < 0.01, ***P < 0.001). (C) The protein expression levels of IKZF3 were assessed in GC cell lines (AGS, NCI-N87, and HGC-27) and the epithelial cell line GES-1. GAPDH was used as a loading control (*P < 0.05, ***P < 0.001). (D) mRNA expression level of IKZF3 determined via qRT-PCR in AGS, NCI-N87 and HGC-27 cells and in GES-1 cells (**P < 0.01, ***P < 0.001).

The RNA and protein levels of *IKZF3* were confirmed in GES-1 cells and three GC cell lines (AGS, NCI-N87, and HGC-27) using immunohistochemistry (IHC), western blot analysis and qRT-PCR. Comparative analysis of IKZF3 expression in four pairs of human GC tissues and adjacent normal tissues revealed significantly increased IKZF3 expression in the cancer tissues ( Figure 2A–D). Thus, these cell lines were selected for further investigation of their biological pathways and functional research.

The relationship between IKZF3 expressions in 80 GC patients was evaluated to investigate the connection between IKZF3 expression and GC prognosis. Furthermore, statistical analysis of clinicopathological data from all the GC patients revealed that high IKZF3 expression was closely linked to tumor size, depth of invasion, lymph node metastasis, distant metastasis, and TNM stage ( *P*  < 0.05). However, no significant associations were found between IKZF3 expression and other clinicopathological features, such as age, sex, Lauren type, histological grade, venous invasion, or nerve invasion ( *P*  > 0.05) ( Supplementary Table S5).

### *IKZF3* knockdown inhibits the proliferation, migration, and invasion of tumor GC cells

**Figure 3:**
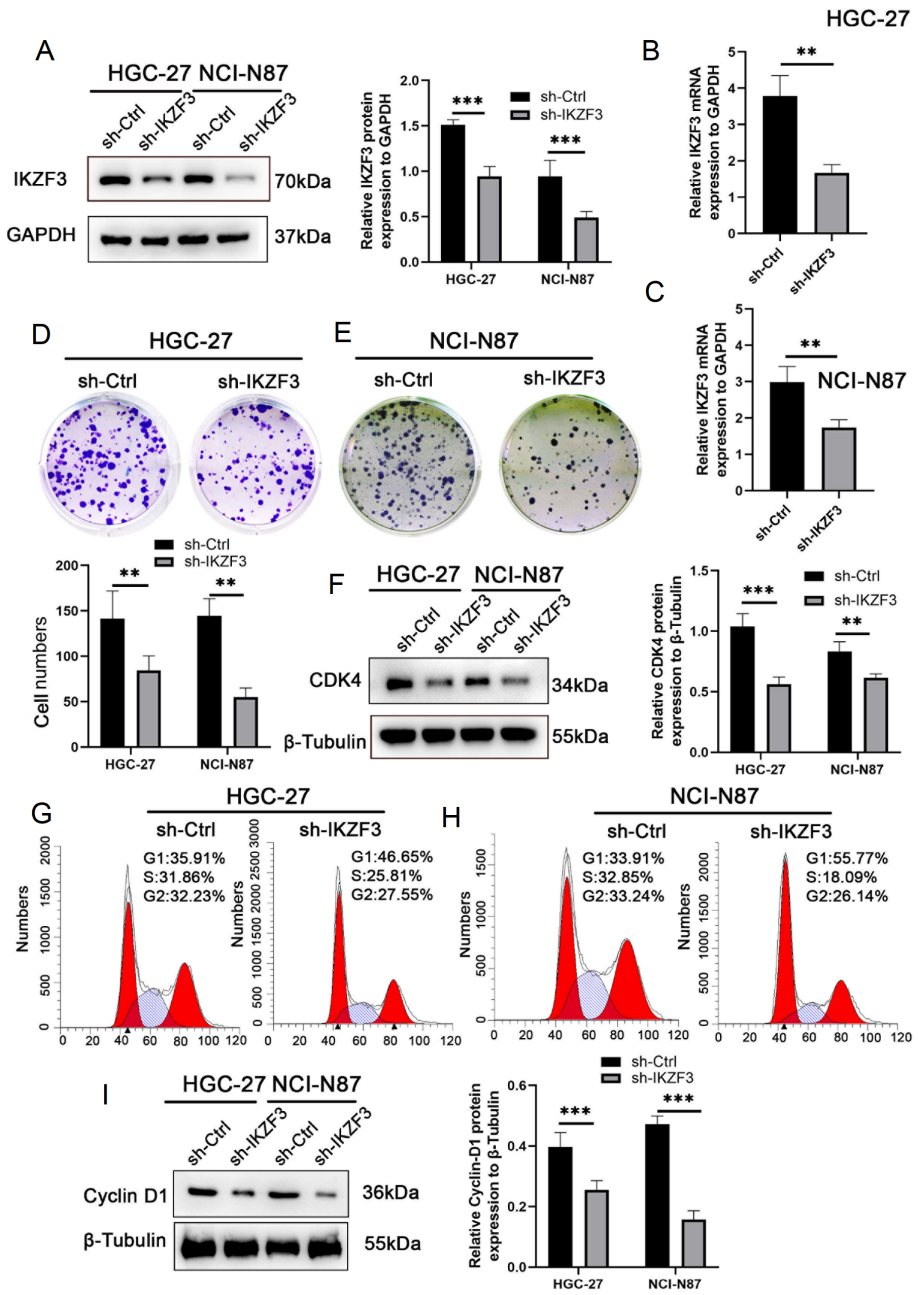
Figure 3 *IKZF3* knockdown effectively suppresses proliferation (A-C) HGC-27 and NCI-N87 cells were transfected with sh-Ctrl and sh-IKZF3, and confirmed by both western blot analysis and qRT-PCR. (D,E) The proliferation capacity of these cells was evaluated through colony formation assays. (F) Proliferation-related proteins, such as CDK4, in HGC-27 and NCI-N87 cells upon IKZF3 knockdown, as confirmed by western blot analysis. (G,H) IKZF3 knockdown influenced the transition from the G1 to S phase in the cell cycle of HGC-27 and NCI-N87 cells, as observed through cell cycle analysis. (I) Proliferation-related proteins, such as Cyclin-D1, in HGC-27 and NCI-N87 cells upon IKZF3 knockdown, as confirmed by western blot analysis. **P < 0.01, ***P < 0.001.

IKZF3 expression was downregulated in HGC-27 and NCI-N87 cells, which presented higher IKZF3 expression than other GC cell lines did. The effectiveness of the knockdown confirmed by qPCR and western blot analysis revealed a significant reduction in IKZF3 levels ( Figure 3A–C). To investigate the impact of *IKZF3* knockdown on the proliferation and cell cycle progression of GC cells, functional experiments were subsequently conducted on these two cell lines.

Cell viability assays revealed that IKZF3 downregulation significantly inhibited the proliferation of HGC-27 and NCI-N87 GC cells ( Figure 3D,E), and western blot analysis revealed decreased expression of CDK4 after *IKZF3* knockdown, indicating that IKZF3 may regulate cell proliferation through this pathway ( Figure 3F).

Flow cytometry was used to assess whether *IKZF3* knockdown induces cell cycle arrest in HGC-27 and NCI-N87 cells. The results showed that *IKZF3* knockdown led to cell cycle arrest at the G1/S phase, effectively inhibiting cell cycle progression ( Figure 3G,H). Western blot analysis confirmed these findings by measuring the expressions of cell cycle-related proteins. Notably, Cyclin-D1, a key regulatory protein involved in cell cycle progression, was significantly downregulated in *IKZF3*-knockdown cells ( Figure 3I). These findings suggest that IKZF3 plays a crucial role in regulating the cell cycle by modulating Cyclin-D1 expression.

**Figure 4:**
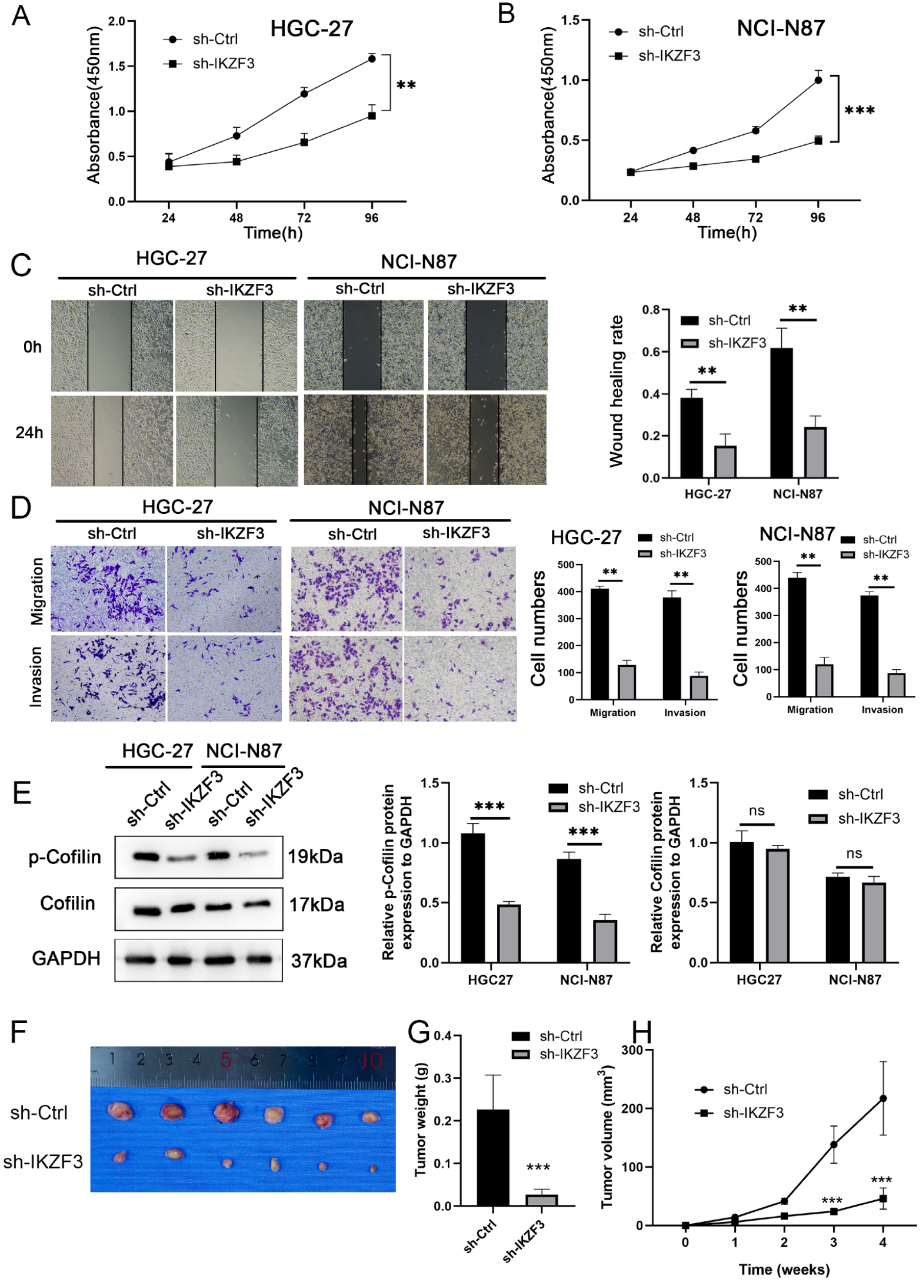
Figure 4 *IKZF3* knockdown inhibits cell proliferation, migration, and invasion (A,B) The proliferation ability of HGC-27 and NCI-N87 cells transfected with sh-Ctrl and sh-IKZF3 was determined by the CCK-8 assay. (C) Wound healing assay examining the mobility of HGC-27 and NCI-N87 cells transfected with sh-Ctrl and sh-IKZF3. (D) HGC-27 and NCI-N87 cells were assessed by the transwell assay for migration and invasion. (E) Migration-related proteins p-Cofilin and Cofilin in HGC-27 and NCI-N87 cells were assessed by western blot analysis. (F) Xenograft models in nude mice were generated via the use of NCI-N87 cells transfected with sh-Ctrl (n = 6) or sh-IKZF3 lentivirus (n = 6), and the silencing of IKZF3 reduced the growth of NCI-N87 tumors in the mice compared with the control group. (G) The tumor weights of the sh-IKZF3 group were significantly lower than those of the vector group. (H) The tumor volumes of the sh-IKZF3 group were smaller than those of the vector group. **P < 0.01, ***P < 0.001.

CCK8 assay was used to examine the ability of IKZF3 to promote the proliferation of HGC-27 and NCI-N87 cells, the results of which were significant ( Figure 4A,B). *IKZF3* knockdown significantly reduced the migratory capacity of the cells in both the wound healing and transwell assays ( Figure 4C,D). The expression of the tumor migration-related molecules Cofilin and p-Cofilin were analyzed by western blot analysis. The results indicated that *IKZF3* knockdown led to decreased expressions of these tumor regulatory molecules ( Figure 4E).

To elucidate the role of sh-IKZF3 in STAD *in vivo*, a xenograft model was established in nude mice. The findings demonstrated that *IKZF3* knockdown significantly slowed tumor growth, resulting in notable reductions in both the tumor mass and volume ( Figure 4F–H).

### *IKZF3* overexpression promotes the proliferation, migration, and invasion of GC cells

**Figure 5:**
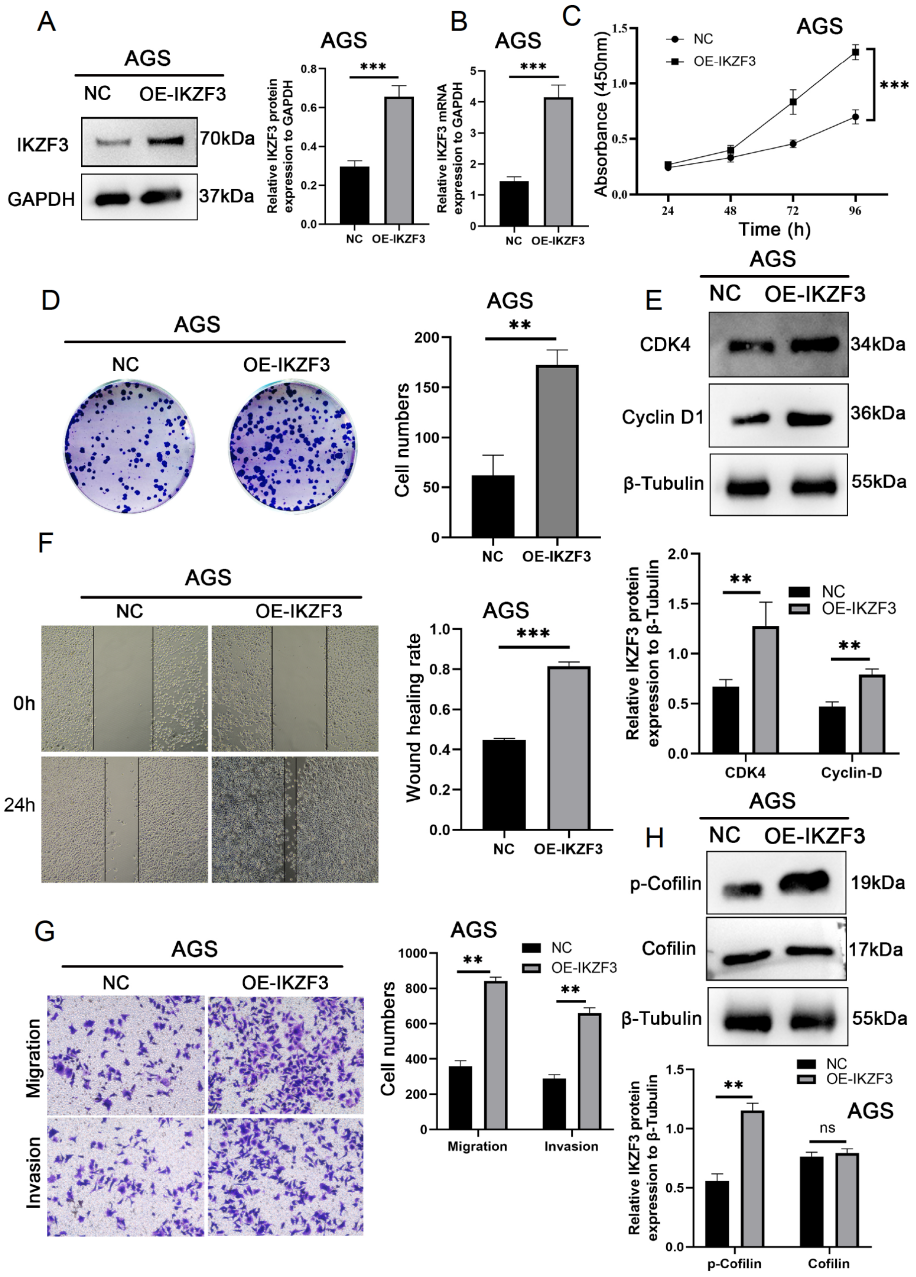
Figure 5 IKZF3 overexpression promotes cell proliferation, migration, and invasion (A,B) The effectiveness of IKZF3 overexpression in AGS cells was assessed by western blot analysis and qRT-PCR. (C,D) The proliferation capacity of AGS cells was ascertained by CCK-8 and colony formation assays. (E) Proliferation-related proteins CDK4 and Cyclin-D1 in AGS cells were evaluated by western blot analysis. (F) Wound healing assay inspecting the mobility of AGS cells. (G) The migration and invasion capabilities of AGS cells were evaluated by transwell assay. (H) Migration-related proteins p-Cofilin and Cofilin in AGS cells were measured by western blot analysis. **P < 0.01, ***P < 0.001 and ns, nonsignificant.

To increase the endogenous expressions of IKZF3 in AGS cells, which presented the lowest IKZF3 expression levels, we transfected the cells with an IKZF3 vector. Following transfection, both the mRNA and protein levels of IKZF3 in AGS cells were significantly elevated ( Figure 5A,B). IKZF3 overexpression markedly increased cell proliferation, as demonstrated by CCK8 and colony formation assays ( Figure 5C,D). Concurrently, the expressions of the proliferation-related proteins CDK4 and Cyclin-D1 were upregulated ( Figure 5E).

Moreover, wound healing and transwell assays revealed that *IKZF3* overexpression significantly enhanced cell migratory capacity ( Figure 5F,G). This finding was further supported by the increased expressions of the migration-related proteins Cofilin and p-Cofilin ( Figure 5H).

### IKZF3 binds to the promoter region of *SMO* to activate the Hedgehog Signaling pathway

**Figure 6:**
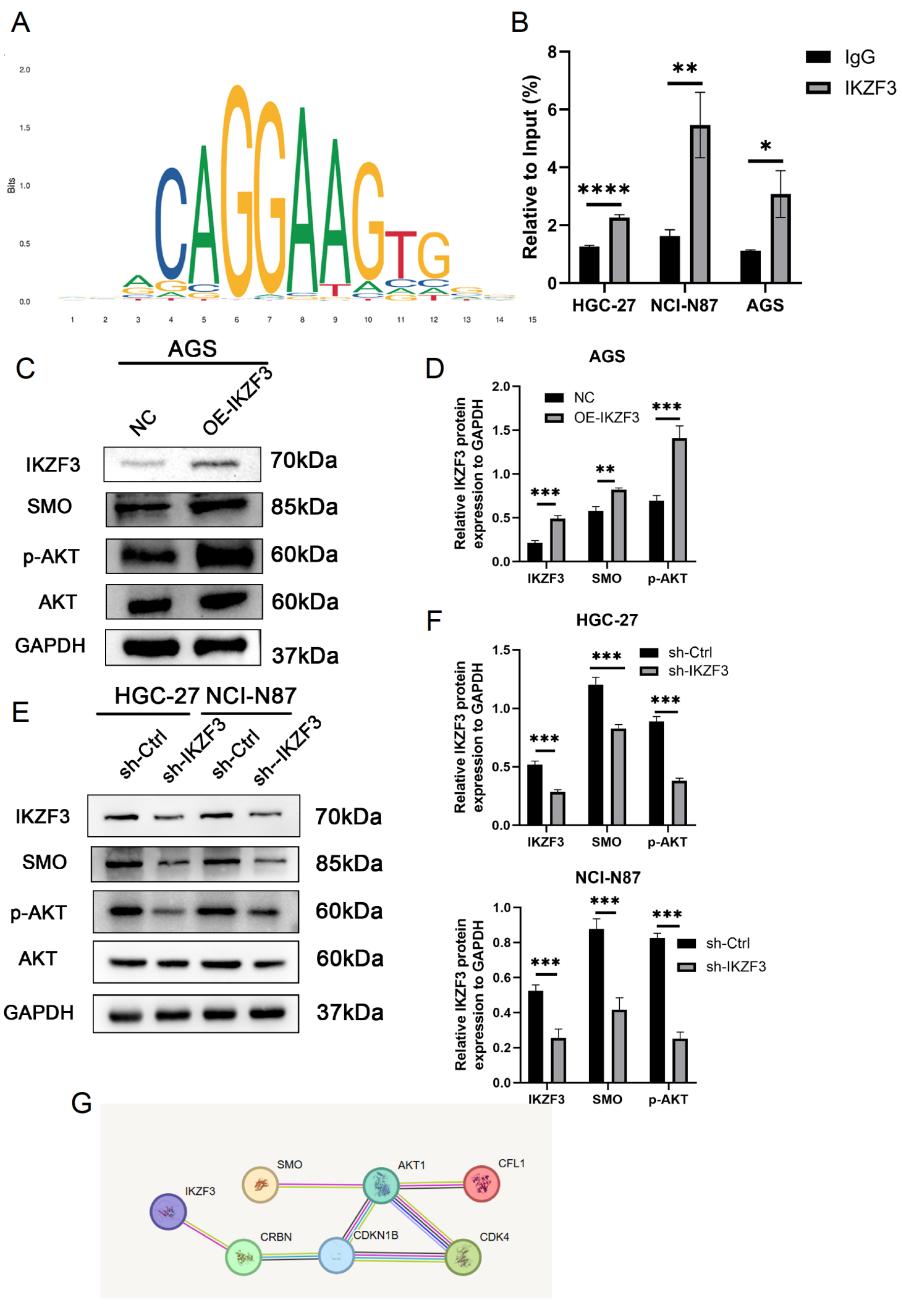
Figure 6 IKZF3 attaches to the promoter region of *SMO* to stimulate the Hedgehog pathway (A) JASPAR database-predicted attachment of IKZF3 with SMO. (B) ChIP analysis in HGC-27, NCI-N87, and AGS cells showed that the SMO promoter region is the primary binding site for IKZF3 (*P < 0.05, **P < 0.01, ****P < 0.0001). (C) IKZF3 overexpression results in increased SMO and p-AKT levels in AGS cells. (D) Western blot analysis was quantified using ImageJ and GraphPad revealed significant differences (**P < 0.01, ***P < 0.001). (E) IKZF3 knockdown results in decreased SMO and p-AKT levels in HGC-27 and NCI-N87 cells. (F) Western blot analysis was quantified using ImageJ and GraphPad revealed significant differences (***P < 0.001). (G) Signaling pathway predicted from data retrieved from the STRING database.

These findings confirmed that IKZF3 regulates SMO expression in GC. To determine whether IKZF3 directly binds to the *SMO* promoter, we identified a binding site using the JASPAR database ( Figure 6A). To identify the binding site, we conducted ChIP analysis in HGC-27, NCI-N87, and AGS cells. Figure 6B shows that the site#1 *SMO* promoter region is the primary binding site for IKZF3 ( Supplementary Table S3).

The protein expression levels of p-AKT and SMO were significantly elevated upon overexpression of IKZF3 in AGS cells ( Figure 6C,D). Knockdown of *IKZF3* in HGC-27 and NCI-N87 cells significantly reduced the protein expression levels of p-AKT and SMO in the Hedgehog signaling pathway ( Figure 6E,F). We utilized the STRING database to conduct a comprehensive pathway analysis, which allowed us to identify and explore the interactions and functional associations among the proteins of interest ( Figure 6G). The findings suggest that IKZF3 creates a positive feedback loop in GC by promoting *SMO* transcription, thereby enhancing the activation of the Hedgehog signaling pathway.

### IKZF3 exerts its biological effects through the Hedgehog signaling pathway

**Figure 7:**
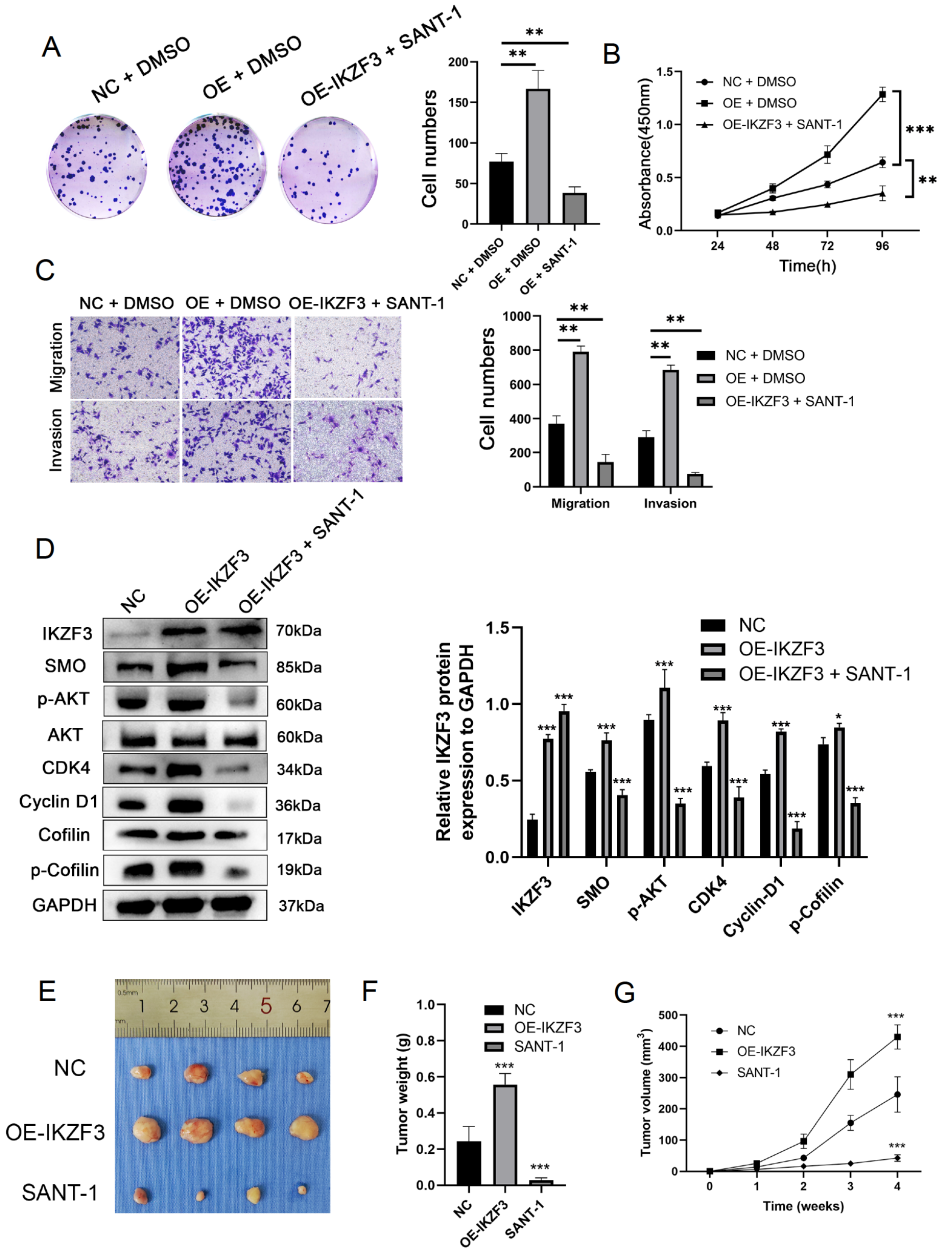
Figure 7 IKZF3 functions via the SMO/AKT signaling (A) The proliferation of AGS cells under SANT-1 treatment was determined via colony formation assay: (i) NC + DMSO, (ii) OE-IKZF3 + DMSO, (iii) OE-IKZF3 + SANT-1. (B) The viability of AGS cells under SANT-1 treatment was determined by CCK8 assay: (i) NC + DMSO, (ii) OE-IKZF3 + DMSO, (iii) OE-IKZF3 + SANT-1; a 10 mM solution prepared by dissolving SANT-1 in DMSO, where the SANT-1 experimental concentration was 2 mM and 0.02% DMSO was used as a negative control. (C) Transwell assay for the migration and invasion ability of AGS cells: (i) NC + DMSO, (ii) OE-IKZF3 + DMSO, (iii) OE-IKZF3 + SANT-1. (D) The levels of IKZF3, SMO, p-AKT, CDK4, Cyclin-D1 and p-Cofilin proteins in AGS cells transfected with NC, OE-IKZF3 and OE-IKZF3 + SANT-1 were determined by western blot analysis. (E) Compared with that in the control group and OE-IKZF3 group, the tumor weight in the SANT-1 group was significantly lower than that in the control and OE-IKZF3 groups. (G)The tumor volume in the SANT-1 group was smaller than that in the control and OE-IKZF3 groups. *P < 0.05, **P < 0.01, ***P < 0.001.

To further validate the involvement of SMO, AKT, CDK4, and Cofilin, we used the SMO inhibitor SANT-1 and overexpressed IKZF3. The results revealed that IKZF3 overexpression increased cell proliferation, migration, and invasion ( Figure 7A–C). In contrast, treatment with SANT-1 significantly decreased these biological behaviors, demonstrating that SANT-1 inhibits the Hedgehog pathway ( Figure 7D). These findings suggest that IKZF3 regulates the expressions of SMO, p-AKT, CDK4, and p-Cofilin. To validate the role of IKZF3 and SANT-1 *in vivo*, we established xenograft models in nude mice. Compared with control treatment, *IKZF3* overexpression (OE-IKZF3) markedly accelerated tumor growth, whereas SANT-1 treatment significantly suppressed tumor mass and volume ( Figure 7E–G).

## Discussion

IKZF3 encodes the zinc finger transcription factor Aiolos, which modulates gene expression through chromatin modifiers [ 18, 19] . The critical role of IKAROS family zinc finger family members (IKZFs) in immunological disorders and malignancies is increasingly recognized [ 20, 21] . Research highlights the involvement of the IKAROS family in carcinoma progression [ 22, 23] . Immune-related genes influence carcinoma prognosis, implicating the immune system in carcinogenesis and metastasis. Evidence supports the potential role of IKZF3 in GC onset and progression.

The *IKZF3* gene is highly expressed in various types of tumors and plays a crucial role in oncogenesis [ 24, 25] . In lung cancer cells, the ectopic expression of Aiolos downregulates several adhesion-related genes, which inhibits cell-to-cell and cell-to-matrix interactions, increases anchorage independence, and promotes cancer proliferation [26]. Additionally, Aiolos overexpression activates the PI3K/AKT Twist axis, enhancing the invasive and migratory capacity of lung cancer cells and increasing cancer stem cell characteristics [27].

Our research provides the first evidence of how IKZF3 transcriptionally regulates the SMO protein and particularly influences the progression of GC. We demonstrated that IKZF3 interacts with the *SMO* promoter region, increasing SMO protein expression and activating the Hedgehog signaling pathway. Additionally, the SMO inhibitor SANT-1 reversed the increase in the proliferation and invasion of GC cells caused by the upregulation of IKZF3. These findings are consistent with those of previous studies on basal cell carcinoma (BCC), pancreatic cancer, and lung cancer, which also highlighted the role of IKZF3 in promoting cancer progression [ 28– 30] .

SMO increases p-AKT activity through unique enzyme recruitment mechanisms [31]. Dysregulation of Hedgehog signaling influences tumor development, metastasis, and drug resistance [ 32, 33] . Hedgehog signaling governs cancer cell proliferation, malignancy, and cancer stem cell (CSC) expansion, driving malignancies [ 34, 35] . Numerous studies have linked the Hedgehog pathway to the maintenance of CSCs in multiple myeloma; chronic myelogenous leukemia; and malignancies of the brain, breast, colon, lung, ovary, pancreas, and prostate [ 36– 41] . SMO plays crucial roles in gastric homeostasis, neoplastic transformation, and epithelial growth. Its abundance in the gastric mucosa highlights its importance in organ development and GC [ 42, 43] .

The SMO complex is broken down, and SMO is reactivated when Sonic Hedgehog signaling is stimulated within the cell. Activated SMO facilitates the translocation of the Gli1 activator to the nucleus, where it activates the transcription of downstream target genes, such as *Cyclin D*, *Bax*, and *Bcl-2*, to regulate cell cycle progression, reduce apoptosis, promote angiogenesis, and ultimately drive tumorigenesis and tumor development [ 44, 45] . Cyclin-D1, a crucial protein in the G1 phase of the cell cycle, binds to and activates the cyclin-dependent kinase CDK4, facilitating the cell cycle transition from the G1 to the S phase and accelerating cell proliferation [ 46, 47] .

The relationship between Cyclin D and CDK4/6 can also be influenced by the PI3K/AKT/mTOR pathway [48]. Cyclin D-CDK4/6 complex phosphorylates retinoblastoma protein, suppressing its growth-inhibitory function and facilitating cell cycle progression. Deletion of *CDKN2A*, which encodes p16 INK4A, is one of the most prevalent anomalies in breast cancer and results in CDK4/6 hyperactivation, driving uncontrolled cell proliferation [49]. Our findings underscore the dual regulatory nature of the Cyclin D-CDK4 axis. Targeting this axis is a promising strategy for cancer treatment, although resistance mechanisms such as noncanonical Cyclin D-CDK2 complex formation and c-Myc amplification can limit its efficacy [ 50, 51] . Targeting the Cyclin D-CDK4 axis could offer promising strategies for cancer treatment.

One of the primary factors affecting cancer progression is the dysregulation of cell migration, which is controlled by multiple oncogenic pathways [ 52, 53] . Most cancer-related deaths are caused by metastasis, a key stage that involves the invasion of cancer cells into adjacent tissues and the vasculature [54]. Cancer cells exhibit cytoskeletal reconfiguration, diminished adhesion function, and loss of cell polarity, among other complex, interdependent dynamic processes [55].

Our results demonstrate the role of p-Cofilin in that it inhibits migration when *IKZF3* is knocked down but promotes migration when IKZF3 is overexpressed, which aligns well with the findings of previous studies [ 56– 58] . Cofilin-1, a nonmuscle isoform of the ADF/cofilin protein family, plays a pivotal role in regulating actin filament turnover both *in vivo* and *in vitro* [59]. Specifically, Cofilin-1 decomposes actin microfilaments and accelerates actin depolymerization, a process that in turn strongly impacts cytoskeletal remodeling [ 60, 61] . Additionally, Cofilin-1 triggers mitochondrial damage, calcium overload, and the initiation of the mitochondrial apoptosis pathway. These effects are dependent on the dephosphorylation of p-Cofilin-1, which facilitates actin depolymerization and mitochondrial translocation [ 62, 63] .

IKZF3 binds to the *SMO* promoter upon Hedgehog pathway activation, promoting GC cell growth and metastasis. Elevated AKT phosphorylation is correlated with increased cancer cell invasion. IKZF3 has biological functions both *in vivo* and *in vitro*. The CDK4-Cyclin-D1 complex controls cell cycle progression by facilitating proliferation. Moreover, p-cofilin-1 inhibits IKZF3-mediated migration by contributing to actin depolymerization and mitochondrial translocation. Future research should elucidate the precise roles of IKZF3 and SMO in GC formation.

The significance of our findings lies in their potential to advance the field of gastric cancer research. By elucidating the role of IKZF3 in tumor biology, we provide a new perspective on the complex network of molecular interactions that drive GC progression. This knowledge could pave the way for the development of novel targeted therapies aimed at inhibiting IKZF3 function, thereby offering a more effective treatment option for patients with advanced GC. Furthermore, our study has several potential clinical implications. The upregulation of IKZF3 in GC tissues suggests that IKZF3 could serve as a biomarker for disease diagnosis and prognosis.

However, we acknowledge that our study has limitations that need to be addressed. One limitation is the relatively small sample size of patient tissues, which may affect the statistical power of our results. Additionally, further mechanistic studies are needed to fully elucidate the downstream signaling pathways regulated by IKZF3 in GC cells. These findings provide a more comprehensive understanding of its role in tumor biology and facilitate the development of targeted therapies.

In summary, IKZF3 regulates GC cell invasion, migration, and proliferation by activating the Hedgehog signaling pathway. SANT-1 counteracts the oncogenic effects of IKZF3 overexpression, inhibiting the enhanced proliferative and invasive capabilities of GC cells, thus highlighting its potential for developing targeted therapeutic approaches against GC.

## Supporting information

25092Supplementary_Tables

## Supplementary Data

Supplementary data is available at *Acta Biochimica et Biophysica Sinica* online.
