# Supplementary material for: IKZF3 promotes gastric cancer progression via Hedgehog signaling activation and is targetable by SANT-1: Role of IKZF3 in gastric cancer
Source: Acta Biochim Biophys Sin (Shanghai). 2025 Aug 4;58(2):369–82. doi: 10.3724/abbs.2025103 (PMC12900698; doi:10.3724/abbs.2025103)
Supplement: 25092Supplementary_Tables [file 25092Supplementary_Tables.docx]

**Supplementary Table S1. Sequences of primers for qRT-PCR**

| Gene | Forward primer (5′→3′) | Reverse primer (5′→3′) |
| --- | --- | --- |
| *IKZF3* | CAACCAGTGGAAAGATGAACTG | CAGTATGGCTTCGCTTATGAAC |
| *GAPDH* | ACGGATTTGGTCGTATTGGGCG | GCTCCTGGAAGATGGTGATGGG |

**Supplementary Table S2. Primary antibodies used in this study**

| Antibody name | Manufacturer | Cat. # |
| --- | --- | --- |
| Anti-IKZF3 | Cell Signaling Technology | #15103 |
| Anti-SMO | Santa Cruz Biotechnology | sc-166685 |
| Anti-AKT | ABclonal | A2696 |
| Anti-p-AKT | ABclonal | AP0637 |
| Anti-Cyclin D1 | Proteintech | 26939-1-AP |
| Anti-CDK4 | Proteintech | 11026-1-AP |
| Anti-CDK6 | Proteintech | 14052-1-AP |
| Anti-Cofilin | Cell Signaling Technology | #5175S |
| Anti-p-Cofilin | Cell Signaling Technology | #3311S |
| Anti-GAPDH | ABclonal | AC001/AC002 |
| Anti-β-Tubulin | ABclonal | A12289 |

**Supplementary Table S3. Sequences of primers for ChIP**

| Sequence Name | Forward primer (5′→3′) | Reverse primer (5′→3′) |
| --- | --- | --- |
| Site#1 | CCCCGGGATACGTTGGAAAA | CTTGCTTGGTCTGAGGTCGT |

**Supplementary Table S4. *SMO* promoter sequence**

| **SMO promoter sequence** |
| --- |
| ttgaggtgagccgagatcacactgctgtgctctgagatcacaccactgtgctccagcctgggcgacagagcgtgactccgtctgaaaaaaaaaaaaaatacctcagactgggtaatttataaagaaaagaagtttaattgggtcacagttcagcaggctatacaggaatcatagcagcttctgctcctggagaagcctcaggaaacttctaatcatggtggaaggcaaagggggagcgagacatcttacatggcaggagcaggaggaagaagcggggaggtgccaaaccctttaaacaatcctcgtgagaactcactcattatcgtgagagcagcacgaggaggatggtgctaaaccattcatgagaaactgcctccgtgatccagttacctcccaccaggcctcacctccaacactgaggattacaattcaacatgagattgggtggggatgcagatccaaaccatatcacctaatattaaccagtaaagattaatatcattcccctagtcaaaactttgagatttccttactatcaagagaattgacccttaattctgtgtgttatttgaggcttcttaccaatcagtttccctacttatctatgtccttattctaactcctcctactcacactttagtcctggattcaaaacccacctcctccacttctatccacactgacctctgtcccctattcctatagcatactcacggtacatctcattttggcagcttatttatcaagtgattactatgtatgctcaagtgaactagatgttttgtgaacactaagagaatgagataaggtccctgacctgaaggagggaacgttgctgttctgtcatgctatgtttcctgagagcaagccatgtatccacaggaaaatggaagtctacaggttcctccaggatgtctctgtgctatgtacatgctggaatttcagaaagacttgctaaattgaattaaattctctctctctctccaattgatatagttgaagtgcaataactaatatgcacttaaactgtccaaagtaacagaactagtccactaccgctaggccatgagcaagtcttccaggggtaagaaaggaaaacccaccctggtgtgcagcccgatcagcaacaaggaaaaagttgctcagtgcaagtctactctcctgtttggagccttgtggttatgctgggttatacttggttgtgcaggtgtaaactgctgaagtgggaaattaagtgtggtgaccaaaagtgtgatgctcaaaggagtgaggaaaggcaaaatgagcagagtggggaagaggagcagcagaaacctcccagagcagatgagcagcagggccatcaaagaaggactggtttccttctcacataaccaaggccagtccctgggaaccacagttcagacttcacatatttgtagcagtaaaggcttgatgttggcaagtttatttcccagacacctgttcccgcaggtaacagcagccagagcagctgccgtgagtgactctgaggtcgtctctctcaaaggtcccgagctggaaggatttggagaagcgcaaaccccaaacccaggttcttctccatggggccccgggatacgttggaaaatctaaccaaagcaggaaaaactggcgggcctggagaacggggcagagatggaacagaagtccccgttgacttcaggcatcccctgtctttcccatcctgagccaggagaaaagtccgcttcctgcagacgacctcagaccaagcaaggtgcccgccgagtctctccttgcaggtccggcccacgatttccactcatctctttcccccgggcgcggggcggcggcaggcggggtcaccagatccccctagcccgggcccctccaggcgccagggacgctgacgctcgcgctcttcctctctcgcctcccctccccacctctccgctccttcgtccagtccctcccccagcctcggcgcaggggggccgggcttggctccgcgaggcccgtgc |

**Supplementary Table S5. Correlations between IKZF3 expression and clinicopathological characteristics in 80 gastric cancer patients**

| Histopathological parameters | Total numbers (n=80) | Expression level of IKZF3 | | *P*-value |
| --- | --- | --- | --- | --- |
|  |  | Low | High |  |
| Age(years)  <65  ≥65 | 29  51 | 13  25 | 16  26 | 0.748 |
| Gender  Male  Female | 32  48 | 13  22 | 19  26 | 0.406 |
| Tumor size  <6  ≥6 | 45  35 | 24  11 | 21  24 | 0.083 |
| Lauren type  Intestinal type  Diffuse type | 56  24 | 23  16 | 33  8 | 0.165 |
| Depth of invasion  T1−T2  T3−T4 | 26  54 | 12  25 | 14  29 | 0.052 |
| Lymph Node metastasis  N0−N1  N2−N3 | 27  53 | 12  17 | 15  36 | 0.031 |
| Distant metastasis  Negative  Positive | 57  23 | 26  9 | 31  14 | 0.004 |
| TNM stage  I−II  III−IV | 23  57 | 15  21 | 8  36 | 0.034 |
| Degree of differentiation  Highly  Moderately and poorly | 47  33 | 17  14 | 30  19 | 0.711 |
| Histological grade  I  II  III | 4  30  46 | 2  13  21 | 2  17  25 | 0.692 |
| Venous invasion  No  Yes | 43  37 | 22  17 | 21  20 | 0.348 |
| Nerve invasion  No  Yes | 42  38 | 19  16 | 23  22 | 0.767 |
